# Premature ejaculation in primary care: communication strategies versus usual care for male patients consulting for a sexual, urogenital or psychological reason – GET UP: study protocol for a cluster randomised controlled trial

**DOI:** 10.1186/s13063-018-2947-2

**Published:** 2018-11-12

**Authors:** Marie Barais, Hélène Vaillant Roussel, David Costa, Jérémy Derriennic, Bruno Pereira, Sébastien Cadier

**Affiliations:** 1 EA 7479 SPURBO, Department of General Practice, Université de Bretagne Occidentale, 22 avenue Camille Desmoulins, 29238 Brest, France; 2 UPU, ACCePPT, Department of General Practice, Faculty of Medicine, Clermont Auvergne University, 28 place Henri Dunant, 63001 Clermont-Ferrand, France; 3 Département Universitaire de Médecine Générale – UFR Médecine Université de Montpellier-Nimes, 2 rue École de Médecine CS 59001, 34060 Montpellier cedex 2, France; 4 Biostatistics unit, Clinical Research and Innovation Department, Clermont-Ferrand University Hospital, 58 rue Montalembert, 63000 Clermont-Ferrand, France; 5 Département de Médecine Générale, UFR Sciences Médicales, Université de Bordeaux, 146 rue Léo Saignat, 33076 Bordeaux cedex, France

**Keywords:** Premature ejaculation, General practice, Sexual dysfunction

## Abstract

**Background:**

Premature ejaculation (PE) is the most common sexual dysfunction among men. According to patients, the general practitioner (GP) is the appropriate professional with whom to discuss this issue. However, few patients receive the medical help needed because GPs find it difficult to talk to their patients about sex. A previous qualitative study provided six strategies described by GPs who had tackled the topic during consultation. A pilot study showed that using one of these strategies after a training course led to an increase in the rate of consultations where the topic was raised: an increase from 6.6 to 30.8%. The aim of this study is to compare whether training in communication skills with these six strategies is more effective than usual care on the incidence of patients bringing up the topic of PE with their GP.

**Methods:**

A cluster randomised controlled trial, stratified over four areas comparing an intervention group, which will receive the six strategies training session, and a control group, which ensures routine medical care. The primary outcome is to investigate the efficacy of a training in communication skills directed towards this pathology, compared with usual care procedures, on the incidence of patients bringing up the topic of PE with their GP. The secondary objective relates to the variation in the quality of life of patients after having recently addressed the topic of PE. Quality of life will be evaluated using the SF-12 health scale, with scoring filled in by the patient immediately after the consultation and 4 weeks later. The patients suffering from PE will be identified if their score is higher than 9 on the Premature Ejaculation Diagnostic Tool filled in 4 weeks after the consultation. The number of patients necessary to highlight a significant difference between the two groups from 5 to 20% is 101. Therefore, a total of 600 patients is expected, 300 in each arm (40 GPs, 15 patients per GP; risk *α* = 5%; power = 90%; intra-cluster correlation coefficient *ρ* = 0.2; Hawthorne effect = 15%; lost-to-follow-up rates for GPs = 10% and for patients = 20%).

**Discussion:**

The implication for practice is the improvement in the quality of patient-centred care within a topic area which encompasses almost 30% of male sex-related complaints.

**Trial registration:**

ClinicalTrials.gov, ID: NCT02378779. Registered on 3 February 2015.

**Electronic supplementary material:**

The online version of this article (10.1186/s13063-018-2947-2) contains supplementary material, which is available to authorized users.

## Background

Many male patients complain about their problems with ejaculation: 21–30% of men, aged between 18 and 80, have admitted suffering from a decrease in, or loss of control of, their ejaculation [1–3]. The quality of life of the patients and their partners is impaired compared with men not suffering from premature ejaculation (PE) [4]. In addition, anorgasmia and low libido, as well as depression and anxiety, are comorbidities significantly associated with PE [5]. Defining PE is not simple: seven to ten different definitions have been proposed since 1970 [6, 7]. The definition chosen by the International Society for Sexual Medicine (ISSM) [8, 9] was an ejaculation which always, or nearly always, occurred prior to, or within about 1 min of, vaginal penetration; the inability to delay ejaculation during all or most vaginal penetrations, and negative personal consequences, such as distress, anxiety, frustration and/or the avoidance of sexual intimacy. Apart from the distinction between acquired and lifelong PE, two other categories have been described: natural variable PE and premature-like ejaculatory dysfunction [10]. Natural variable PE corresponds to the normal variation in sexual performance. The premature-like ejaculatory dysfunction corresponds to the patient’s distorted perception of time before ejaculation: the patient is convinced that he is suffering from PE although his Intravaginal Ejaculation Latency Time (IELT) is more than 1 min. While the prevalence, according to men’s self-reports, is 30%, the rate drops to 3% when the time factor is considered in isolation [5]. This statement illustrates the incompatibility between the academic definition and the actual complaints from patients.

One study showed that most of the men interviewed anonymously, in their general practitioner’s (GP’s) waiting room, considered it important to talk with their GP about their sexual concerns [11]. Almost half of them preferred that their GP initiate any discussions about sexuality. More than two thirds of the respondents would have liked their GP to signal their open-mindedness by directly addressing sexual topics during the consultation [11]. While 80% of men who participated in the study suffered at least occasionally from a reported sexual problem, only 12% had already consulted their GP about this [11]. The guideline of the ISSM emphasised the role of GPs in PE care [10]. The GP is the first medical professional that the patient meets to discuss his problem. The main objective of the GP is ‘to recognise PE and make the patient feel comfortable about getting help’ [10]. The physician-patient relationship, developed during primary care consultations, is a foundation of patient-centred care. After having identified the problem, those GPs who had good communication skills sometimes initiated treatment. However, several studies have described the difficulties reported by GPs in talking about sex. Lack of time is the most significant factor [12–14]. Sexual dysfunction is often considered to be of secondary importance after information and detection of sexually transmitted infections, contraceptive counselling, vaccinations, etc. [12–15]. In addition, it is difficult for GPs to deal with such a time-consuming topic. Feeling that they have insufficient expertise, or are ill-qualified to deal with sexual problems, are also contributing factors [13, 16]. The lack of training in sexology during their academic studies was highlighted [13, 16]. The average of 3 hours’ tuition was not sufficient to teach all the pathologies and their management [13, 16]. ‘Opening a can of worms’ is the expression often used by GPs to describe the difficulties they feel in addressing this kind of pathology during consultation [13, 16]. Factors affecting the GPs’ involvement are known: training in communication skills was the most important predictor for sexual-history taking [17].

Several types of treatment, both pharmacological and psychological, now exist and have been evaluated and recommended: PE is a treatable pathology [10, 18–21].

The first study on this topic occurred in 2009 with a qualitative study to bring to the fore the strategies used by GPs to initiate the discussion on PE. Eleven GPs participated in semi-structured interviews composed of open-ended questions for exploration and closed questions to refine the participants’ answers [22]. According to the content analysis of the interviews transcribed verbatim, six different strategies used by the GPs to tackle the subject were identified (described in the ‘Methods’ section).

The main aim of this study is to investigate whether training in communication skills with these six strategies is more effective than usual care on the incidence of patients bringing up the topic of PE with their GP.

## Methods

### Outcome

The primary outcome is the incidence of patients bringing up the topic of PE with their GP.. This was undertaken in the group where the GPs received training in communication skills directed towards this pathology, compared with the patients who received the usual care procedures.

The secondary outcome relates to the variation in the quality of life of patients after they have addressed the topic of PE as measured by the SF-12 questionnaire.

### Trial design

**Fig. 1:**
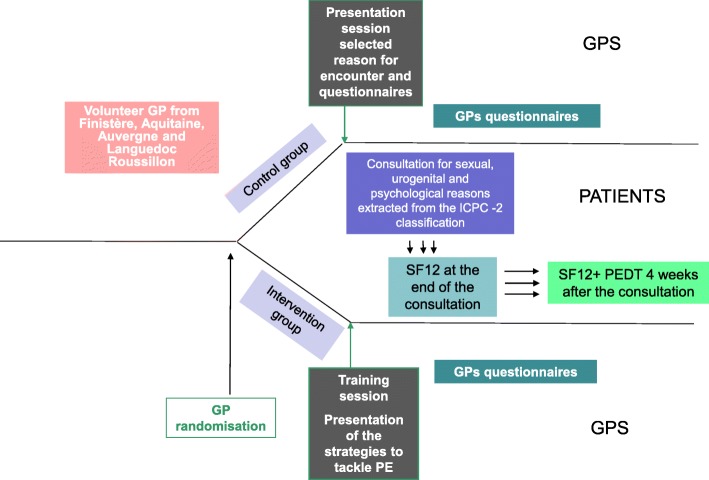
Study design

**Fig. 2:**
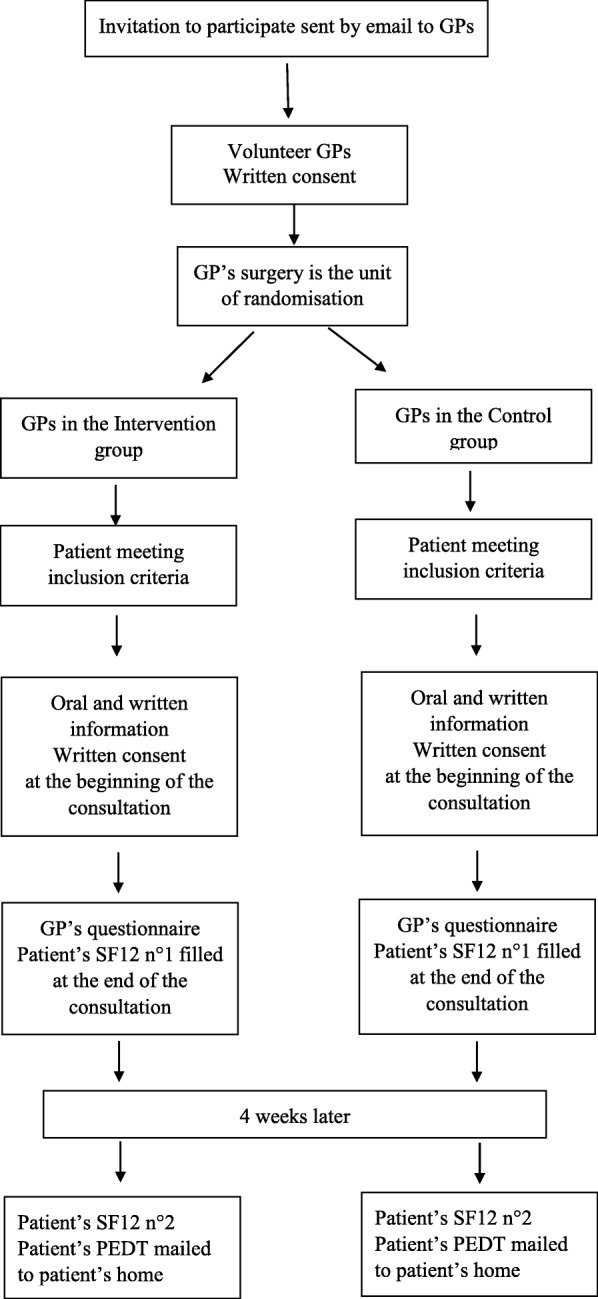
Participant timeline

**Fig. 3:**
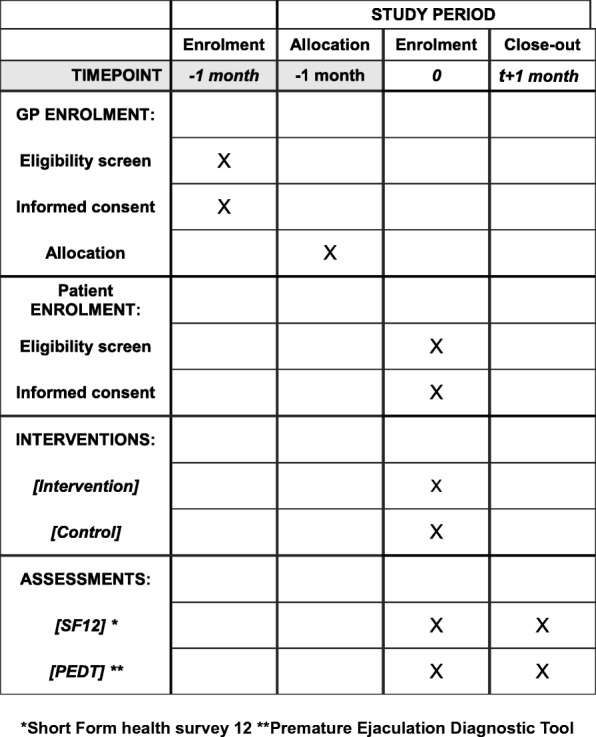
Standard Protocol Items: Recommendations for Interventional Trials (SPIRIT) Schedule of study procedures

The GET UP trial is designed as a two-arm, randomised controlled trial with two parallel clustered groups assigned to an intervention group (IG) or a control group (CG) stratified by region (Figs. 1, 2 and 3).

### Study setting

GPs from four French regions will participate: Brittany, Aquitaine, Massif Central, and Languedoc-Roussillon-Midi-Pyrénées. In each of the four regions, a leading investigator will be identified, to be responsible for recruitment of GPs, identification, data collection and completion of CRFs, along with following up study patients and adherence to study protocol.

The list of study sites can be obtained from the Direction de la Recherche Clinique et de l’Innovation at Brest. The GPs will not be incentivised to take part.

### Participants, interventions, and outcomes

#### Eligibility criteria

##### Patient inclusion criteria

- Consecutive male patients, between 18 and 80 years old, and consulting for a sexual, urogenital or psychological reason will be included. We added age limits to the registered protocol because, above 80 years of age, the specificity of the care required for those of advanced age was out the scope of the study. A list of sexual, urogenital, and psychological reasons extracted from the International Classification of Primary Care, Second edition (ICPC -2) classification will be provided for the GPs to guide their inclusions. The GPs will inform the patient of the study after knowing the reason for the encounter, at the beginning of the consultation. The key information is written on a consent form where the study on sexual dysfunction is described and where it is explicitly written that the patient agrees to participate in the study. The patient will sign the document after he has been given the explanations. The participants will not be incentivised to take part.

##### Patient non-inclusion criteria

Patients consulting for reasons other than sexual, urogenital or psychological will not be included. Patients consulting for one of the required reasons but who are unable to speak French, patients with psychiatric disorders affecting judgement and patients unable to sign an informed consent form were excluded.

##### GP inclusion criteria

The volunteer GPs are involved in the Department of Primary Care as trainers. GPs and patients from these offices are representative of the French population consulting in primary care [23].

##### GP non-inclusion criteria

GPs with an exclusive specialty (e.g., acupuncture, homeopathy) and physicians who have received specialised training in sexology and in communication skills during specific training or dedicated tuition will not be included in the sample of GPs.

#### Intervention

##### Intervention

The intervention will consist of a training session with the aim of directing the GPs to use the strategies found during the qualitative part of the pilot study. At an interactive 4-h workshop, GPs assigned to the IG will be trained to handle cases. The session will begin with a brainstorming session to bring out the GPs’ knowledge of PE and any questions that they have on the subject. This will be followed by a presentation which will describe PE, its different types and definitions, the physiopathology and aetiology of PE, and the treatments available [8]. These theoretical elements will be presented through a slide presentation, designed and approved by all the committee members. The chosen definition includes the three criteria of the ISSM (time, loss of control, and negative personal consequences) without mentioning in detail the ‘1-minute’ factor [6]. The results of the qualitative phase will be explained and the different strategies will be explicitly defined to provide operational and usable approaches to communication [22]. The six strategies were divided into two sections:

three attitude-related strategies for revealing the problem:

- Being particularly receptive to the patient throughout the consultation: especially at the end when the physician was about to open the door, in order to create a gap that the patient could use to express his problem. The physician consciously creates an attentive environment, using non-verbal behaviour which will encourage the patient to speak freely
- Using humour: lightening the atmosphere by using gentle humour without being vulgar or insensitive
- Taking a matter-of-fact approach to play down any embarrassment in the patient’s view of sexuality: emphasising the natural and mechanical function of sexuality allows the GP to play down the emotional aspect of the subject
- Three investigative strategies with interviews deliberately directed towards sexual dysfunction:
- Systematic questioning during consultations dedicated to prevention
- Symptoms that the patient could be experiencing: the GP suggests a list of signs and symptoms connected to the current clinical situation, for instance: ‘you’re presenting symptoms of depression, and depression may bring on physical, psychological, and even sexual tiredness’. The patient chooses one of these to talk about his PE
- Facilitating the patient’s verbal expression, enquiring about the patient’s psycho-social and medical history and his daily environment enables the subject of PE to be raised

The training session will be provided by the Steering Committee member in each of the four regions. Their expertise is as follows: three have had specialised training in sexology and three have had specialised training in education. They will receive audio-visual training in the presentation of the slides to ensure the replication of the intervention. GPs will be trained, with practice situations, using role-play performed with the committee member and the other GPs. Available treatments, based on the ‘guidelines for the diagnosis and treatment of premature ejaculation’ [8] will be presented during the session. The session will end with the presentation of the outcome questionnaires.

The intervention session will follow this agenda:

- 30 min will be devoted to brainstorming
- 1 h to the presentation of theoretical elements, and
- 2 h will be devoted to role-play practice situations

The intervention will occur at the GP’s surgery or during a professional meeting, depending on the choice of the GP.

##### Usual care

GPs registered in the CG will provide care according to their usual practices. The definition of usual care described here is ‘the clinical care without any value judgment’ and centred on the patient [24]. We concentrated on reducing influences on the control GPs in the information session and through the GP questionnaire [25]. The GPs in the CG will attend a 2-h information session to learn about the case report forms and the patients’ inclusion/exclusion criteria. The session will be provided by the Steering Committee member in each of the four regions. A slide presentation, designed and approved by all the committee members, will be presented along with the reasons for the consultation and the GP questionnaire to be completed.

#### Data collection

##### Outcomes and measurements

To measure the incidence of patients suffering from acquired and lifelong PE, natural variable PE and premature-like ejaculatory dysfunction, the final diagnosis of PE will be made by referring to the answers given to the Premature Ejaculation Diagnostic Tool (PEDT) sent back by the patient 4 weeks after the consultation (please refer to the ‘Measurements’ section). The patients suffering from PE will be identified if their score is higher than 9 on the PEDT. The PEDT questionnaire is an extensively validated, self-report measure that can systematically assess DSM-IV-TR criteria to provide accurate diagnoses of PE/no-PE [26]. It is a screening tool with cut-off scores, brief and easy to administer, and recommended by the International Society of Sexual Medicine [8, 26, 27]. The self-report questionnaire, in our opinion, seems to handle delicate, intimate questions with appropriate sensitivity. We followed a linguistic and cultural validation procedure to obtain a French version of the questionnaire. Additional file 4 is an abstract describing the procedure we followed.

Patients in the two groups will have to complete a self-report questionnaire on quality of life, the SF-12 questionnaire, in a French-validated version right after the consultation, and again, 4 weeks after the consultation [28]. The SF-12 questionnaire right after the consultation will be available in the waiting room. The SF-12 questionnaire, 4 weeks after the consultation, will be posted or e-mailed to the patient. The SF-12 is a self-report questionnaire exploring quality of life [28]. The original questionnaire contained 36 items divided into eight scales, the SF-36 [28]. The SF-12 was created to shorten the responses and included one or two items from each of the eight SF-36 scales. The French version of the SF-12 is correlated to the SF-36 [28]. We chose a general quality of life self-report questionnaire for several reasons. A generic scale was useful to avoid influencing patients in the CG. The availability of a validated French version was another argument supporting the choice of this scale [28].

### Feasibility

As far as we are aware, this interventional multicentre study will be the first one concerned with PE in the area of primary health care. We created a pilot intervention study in primary care in order to estimate the feasibility of such a study design, as well as the incidence of diagnosed PE with, and without, the use of strategies. Thirteen GPs agreed to participate in the IG, six of whom included their patients in the study. Twenty-four GPs participated in the CG, 13 of whom included their patients in the study. The GPs in the IG included 26 of their patients in the study. They identified eight patients suffering from PE: 30.8%. The GPs in the CG included 61 of their patients. Among them, four were suffering from PE: 6.6% (*p* value = 0.004, OR = 6.6768, 95% confidence interval (CI) (1.55; 34.40)).

### Sample size estimation

As this study had been designed as a cluster randomised trial, a sample size estimation was proposed which would highlight a significant difference between the two randomised groups in terms of the incidence of new cases of patients bringing up the topic of PE with their GP. It takes into account an intra-cluster correlation coefficient (ICC) varying between 0.05 and 0.20 and includes a recruitment of around 15 patients per GP [29]. In order to take into account between- and within-GP variability, expressed by intra-class correlation coefficient (ICC), this sample size must be increased by an inflation factor: 1 + (*m* − 1). ICC, with *m* the (mean) number of patients included by each GP. According to the literature [29, 30], it is not so easy to determine the appropriate value of ICC. It depends of many factors. We have chosen to fix ICC between 0.05 and 0.20, according to previous references. With 15 patients included by each GP, sample size estimation was 360 for ICC equals 0.05 and 800 for ICC at 0.20, and around 960 for ICC at 0.25. Furthermore, it is usual to observe a Hawthorne effect in this type of study. In our feasibility study, the incidence of patients who brought up PE with their GP in the CG was 6.6%. Following the guideline of PE updated by the ISSM, ‘5% of the population have an ejaculation latency of less than 2 minutes’ [8]. We estimated the incidence in the CG at around 5%. Usually in individual randomisation, *n* = 101 patients in total is necessary to highlight an absolute difference between 5 and 20%. For a two-sided type I error, *α* equals 0.05 and has a statistical power of 90%. Also, taking these aspects into account 600 patients, recruited by 40 GPs, must be included and then divided into 300 patients in each arm.

#### Recruitment

All the GPs will receive an e-mail presenting the study as a work on the topic of ‘sexual dysfunction’. Volunteer GPs will be recruited. We asked the GPs from both groups at the beginning of the study whether they had ever benefited from specific training in communication skills before this study. This specific question will allow us to evaluate whether GP group differences result from the training versus no training conditions. The cluster will be identified before the randomisation. All the participants will receive a certificate at the end of the ‘usual care’ and ‘intervention’ sessions. The training sessions and workshop will take place within a suitable schedule for the GPs involved, in a pleasant and constructive atmosphere. One phone call per month, and two e-mails per month will be made to remind the GP participants to include patients. The slides presented in both the CG and the IG will be at the GPs’ disposal after the sessions. In each communication, the research team will insist on knowing the possible issues encountered by the GPs throughout the entire process. The enrolment period will extend over 12 months.

### Assignment of interventions (for controlled trials)

#### Allocation

The randomisation is stratified by region, and by physician’s gender, using a random block randomisation sequence generated in Stata software (version 13, StataCorp, College Station, TX, USA). The GP’s surgery is the unit of randomisation. A GP investigator and their patients will be assigned to the same group. The patients will not know to which group their GP has been randomised. Investigators from the same GP’s surgery are randomised within the same group in order to avoid contamination bias.

The participating GPs will be randomised using Stata version 13 (StataCorp, College Station, TX, USA) which is an online, central randomisation service. Allocation concealment will be ensured as the service will not release the randomisation code until the GP has been recruited into the trial in each region.

#### Blinding

Due to the nature of the intervention, neither the GPs from the intervention team nor the researchers recruiting the GPs can be blinded to allocation, but it will be strongly impressed upon them that they must not disclose the allocation status of the participants at the follow-up assessments. The objective of this measure is that a GP from the CG should not know that they are in a CG. An employee outside the research team will collect the questionnaires (SF-12 and PEDT) and feed data into the computer on separate datasheets so that the researchers can analyse data without having access to information about the allocation. The patients will be blinded to the allocation of their GP and the trial aim.

### Data collection, management, and analysis

#### Data collection methods

The questionnaires filled in by the GPs and the patients will be mailed to the main centre in Brest. The data from these questionnaires will be implemented in an Excel® table. The data collection will be stored at the Brest centre.

A letter will be sent to the patients to remind them to mail the second SF-12 questionnaire and the PEDT questionnaire if they have not returned them 5 weeks after the consultation.

#### Data management

Original study forms will be entered and kept on file at the participating site. A subset will be requested later for quality control; when a form is selected, the participating site staff will access that form, copy it, and send the copy to the Data Coordinating Centre.

Participant files are to be stored in numerical order and stored in a secure and accessible place and manner. Participant files will be maintained in storage for a period of 15 years after completion of the study.

#### Statistical methods

Analyses will be performed using Stata software, version 13 (StataCorp, College Station, TX, USA). All data will be analysed on intention to treat. The tests will be two-sided, with a type I error set at *α* = 0.05. Baseline characteristics (GPs and their patients) will be presented for each randomisation group as the mean ± standard deviation (SD) or the median (interquartile range) according to statistical distribution for continuous data and according to the number of patients and associated percentages for categorical parameters.

The comparisons between the randomised groups will be carried out using the Student *t* test or the Mann-Whitney test where appropriate, (1) normality verified by the Shapiro-Wilk test and (2) homoscedasticity by the Fisher-Snedecor test for quantitative parameters and using chi-squared or Fisher’s exact tests for categorical variables.

Outcomes will pertain to the cluster level using usual statistical tests and with more specific approaches at the individual level. Hierarchical regression models (generalised linear mixed model due to binary endpoint) will be performed as primary analysis: (1) to estimate the effect of intervention on the incidence of new cases of patients suffering from acquired and lifelong PE, natural variable PE and premature-like ejaculatory dysfunction and (2) to take into account the variability between and within, the GP cluster groups. Intra-class correlation coefficients will be presented by arm with a 95% CI. Then, GP characteristics (gender, age, geographical area) or patient characteristics (age, socioeconomic status) should be considered as covariate (fixed effects in mixed models) in adjusted multivariable analyses. The adjusted and unadjusted results will be presented: absolute numbers of patients who have raised the topic of PE with their related GP, relative risks and 95% CIs.

For secondary endpoints, comparisons at the individual level between randomised groups will be performed using an analysis of covariance for correlated data with the baseline score as a covariate, as suggested by Klar and Darlington [31] for the SF-12 quality of life scores. Results will be expressed as effect-sizes and 95% CIs. The PEDT will be analysed as a quantitative score using statistical methods described for the SF-12 quality of life and as a binary outcome (< ≥ 11) with the use of a generalised linear mixed model.

A sensitivity analysis will be carried out to study the attrition bias and to characterise the statistical nature of missing data in order to propose the most appropriate missing data imputation method. More precisely, a maximum bias approach will be performed for the primary outcome and a multiple imputation method for secondary outcomes.

The analysis of the type of strategies used by the GPs in the IG will be descriptive and exploratory.

### Monitoring

In each of the four regions, a Steering Committee member will have participated in the design of GET UP and will review the progress of the study and, if necessary, will agree changes to the protocol. A data monitoring committee is not needed here as it is a low-risk trial, not involving any pharmaceutical substances.

An interim analysis is performed on the primary endpoint when 50% of patients have been randomised and have completed the SF-12 and the PEDT 1 month after the inclusions. The interim analysis will be performed by the statistician, blinded to the treatment allocation. The statistician will report to the data manager. The data manager will have unblinded access to all data and will discuss the results of the interim analysis with the Steering Committee in a joint meeting.

The Steering Committee will decide on the continuation of the trial and will report to the Central Ethics Committee. The Steering Committee will also declare the end of the study. It may decide to stop the study if the inclusion rate is too weak or for administrative reasons.

#### Harm

In our study, no drugs will be used. No adverse outcome is defined.

#### Auditing

A clinical research associate commissioned by the promoter will ensure the successful completion of the study, the collection of data generated in writing and documentation, and will record and report in accordance with the standard operating procedures of the DRCI Brest and in accordance with good clinical practice and the laws and regulations in force.

The investigator and his team members agree to make the data available during the quality control visits at regular intervals by the clinical research associate. During these visits, the following items will be reviewed:

##### Informed consent

- Compliance with the study protocol and procedures defined therein
- Quality of data collected in the case report: accuracy, missing data, data consistency with the documents ‘source’ (medical records, appointment books, etc,)

Moreover, investigators undertake to accept quality assurance audits by the promoter as well as inspections by the competent authorities. All data, all documents and reports may be subject to audits and regulatory inspections.

## Discussion

This intervention study will be the first concerned with PE in the area of primary health care. Six different strategies for tackling the subject were brought to light after a qualitative phase with GPs was compared with usual care procedures in primary health care. A French version of the Premature Ejaculation Diagnostic Tool will be used to detect patients from the IG and the CG who suffer from PE.

One major limitation is the unblinded GPs in the IG. As the intervention is based on training in strategies to tackle PE, it was impossible to leave them blind to the main aim of the study. The evaluation of the diagnosis of PE will be made by the GP. This limitation is counterbalanced by the fact that the PEDT sent after 1 month will also contribute to making the positive or negative diagnosis of PE.

We selected our participants via an e-mail, mentioning a study on ‘sexual dysfunction’ at the first step of the study. We wanted to inform them about the topic raised during the intervention. The GPs in the CG knew that the study was investigating sexual dysfunction: this could lead to increased awareness of sexual dysfunction, including PE, and thereby lead to an increase in the rate of consultations where PE was raised. We did not mention ‘sexual dysfunction’ on any other occasion to the CG.

The definition of PE references a pathology concerned with ‘vaginal penetration’. However, we want to enlarge the study to include men with a different sexual orientation. We did not include any questions about heterosexual or homosexual orientation in our questionnaires.

The investigation of an intervention involving six communication strategies and the development of a sexual health communication tool for use in primary care would meet the needs of both practitioners and patients [32]. Implementing the strategies in a real practice context is the main added value of this trial. The ISSM guideline states that GPs have an important role to play in the diagnosis and treatment of PE [9]. This trial provides a pragmatic way to help the GPs to do this. The chosen format of an intervention lasting 4 h, and the rapid questionnaire to complete, were adapted to the time requested by the study and available for the GPs. A cluster design was chosen for pragmatic reasons and to avoid contamination bias. Doing pragmatic research in real practice is quite unusual for French GPs. The strategies are not exclusive to PE and may be useful for initiating discussion of other crucial issues related to sensitive topics. This concrete step of ‘how to do that in practice and how to be sure that it is efficient’ is often lacking in primary care. We wanted to connect research and practice in order to make the conclusions helpful for patients.

### Trial status

The study has not completed patient recruitment at the time of submission.

The Standard Protocol Items: Recommendations for Interventional Trials (SPIRIT) Checklist is included as Additional file 1. The Consolidated Standards of Reporting Trials (CONSORT) Checklist is incuded as Additional file 2.The Template for Intervention Description and Replication (TIDieR) Checklist is incuded as Additional file 3. The abstract of the linguistic and cultural validation procedure to obtain a French version of the Premature Ejaculation Diagnostic Tool (PEDT) is incuded as Additional file 4.

## Additional files

Additional file 1: Standard Protocol Items: Recommendations for Interventional Trials (SPIRIT) Checklist. (DOC 120 kb)

Additional file 2: Consolidated Standards of Reporting Trials (CONSORT) Checklist. (DOCX 30 kb)

Additional file 3: Template for Intervention Description and Replication (TIDieR) Checklist. (DOCX 29 kb)

Additional file 4: Abstract of the linguistic and cultural validation procedure to obtain a French version of the Premature Ejaculation Diagnostic Tool (PEDT). (DOCX 14 kb)

## Abbreviations

CG: Control group
CPP: Comité de Protection des Personnes
GP: General practitioner
IG: Intervention group
ISSM: International Society of Sexual Medicine
PE: Premature ejaculation
PEDT: Premature Ejaculation Diagnostic Tool
