# Supplementary material for: Premature ejaculation in primary care: communication strategies versus usual care for male patients consulting for a sexual, urogenital or psychological reason – GET UP: study protocol for a cluster randomised controlled trial
Source: Trials. 2018 Nov 12;19:622. doi: 10.1186/s13063-018-2947-2 (PMC6233366; doi:10.1186/s13063-018-2947-2)
Supplement: Supplementary file 4 — Abstract of the linguistic and cultural validation procedure to obtain a French version of the Premature Ejaculation Diagnostic Tool (PEDT). (DOCX 14 kb) [file 13063_2018_2947_MOESM4_ESM.docx]

Linguistic and cultural validation procedure to obtain a French version of the PEDT

Introduction: Many male patients complain about their ejaculation: 21-30% of men aged between 18 and 8059 have admitted suffering from a decrease in, or loss of control of, their ejaculation. A qualitative study took place in 2009 to bring to the fore 6 strategies used by GPs to initiate the discussion on premature ejaculation. The Get Up project aims to investigate whether training in communication skills specific to this topic would be more effective than the usual techniques doctors employ to encourage patients to speak freely about their complaints of premature ejaculation. The PEDT questionnaire is an extensively validated, self-report measure that can systematically assess DSM-IV-TR criteria to provide accurate diagnoses of PE/no-PE. The five questions of the questionnaire are a screening tool with cut-off scores, brief and easy to administer and recommended by the International Society of Sexual Medicine. The aim of this study was to translate the PEDT into French following a cultural and linguistic procedure.

Method: Forward and backward-translations with professional native French and English translators were created first. Then the resulting French version was submitted to 17 bilingual or fluent English speaking GPs using a Delphi procedure.

Results: After three Delphi rounds, French GPs agreed on the French version of the Five questions of the PEDT.

English version of the PEDT

1) How difficult is it for you to delay ejaculation?

2) Do you ejaculate before you want to?

3) Do you ejaculate with very little stimulation?

4) Do you feel frustrated because of ejaculating before you want to?

5) How concerned are you that your time to ejaculation leaves your partner unfulfilled?

French version of the PEDT

La version finale du questionnaire diagnostic de l’éjaculation prématurée après 3 rondes de Delphi était la suivante :

1) A quel point vous est-il difficile de retarder votre éjaculation ?

2) Vous arrive-t-il d'éjaculer avant que vous ne le souhaitiez ?

3) Une très légère stimulation suffit-elle à vous faire éjaculer ?

4) Vous sentez vous frustré d’éjaculer avant de l’avoir décidé ?

5) A quel point vous sentez-vous préoccupé par l'insatisfaction de votre partenaire liée à votre délai avant éjaculation ?
